# The Opportunities and Challenges regarding Induced Platelets from Human Pluripotent Stem Cells

**DOI:** 10.1155/2021/5588165

**Published:** 2021-05-01

**Authors:** Meng-Xue Xu, Li-Ping Liu, Yu-Mei Li, Yun-Wen Zheng

**Affiliations:** ^1^Institute of Regenerative Medicine, Affiliated Hospital of Jiangsu University, Jiangsu University, Zhenjiang 212001, Jiangsu Province, China; ^2^School of Biotechnology and Health Sciences, Wuyi University, Jiangmen 529020, Guangdong Province, China; ^3^Department of Gastrointestinal and Hepato-Biliary-Pancreatic Surgery, University of Tsukuba Faculty of Medicine, Tsukuba, Ibaraki 305-8575, Japan; ^4^Yokohama City University School of Medicine, Yokohama, Kanagawa 234-0006, Japan

## Abstract

As a standard clinical treatment, platelet transfusion has been employed to prevent hemorrhage in patients with thrombocytopenia or platelet dysfunctions. Platelets also show therapeutic potential for aiding liver regeneration and bone healing and regeneration and for treating dermatological conditions. However, the supply of platelets rarely meets the rising clinical demand. Other issues, including short shelf life, strict storage temperature, and allogeneic immunity caused by frequent platelet transfusions, have become serious challenges that require the development of high-yielding alternative sources of platelets. Human pluripotent stem cells (hPSCs) are an unlimited substitution source for regenerative medicine, and patient-derived iPSCs can provide novel research models to explore the pathogenesis of some diseases. Many studies have focused on establishing and modifying protocols for generating functional induced platelets (iPlatelets) from hPSCs. To reach high efficiency production and eliminate the exogenous antigens, media supplements and matrix have been optimized. In addition, the introduction of some critical transgenes, such as *c-MYC*, *BMI1*, and *BCL-XL*, can also significantly increase hPSC-derived platelet production; however, this may pose some safety concerns. Furthermore, many novel culture systems have been developed to scale up the production of iPlatelets, including 2D flow systems, 3D rotary systems, and vertical reciprocal motion liquid culture bioreactors. The development of new gene-editing techniques, such as CRISPR/Cas9, can be used to solve allogeneic immunity of platelet transfusions by knocking out the expression of *B2M*. Additionally, the functions of iPlatelets were also evaluated from multiple aspects, including but not limited to morphology, structure, cytoskeletal organization, granule content, DNA content, and gene expression. Although the production and functions of iPlatelets are close to meeting clinical application requirements in both quantity and quality, there is still a long way to go for their large-scale production and clinical application. Here, we summarize the diverse methods of platelet production and update the progresses of iPlatelets. Furthermore, we highlight recent advances in our understanding of key transcription factors or molecules that determine the platelet differentiation direction.

## 1. Introduction

In mammals, platelets are produced by mature megakaryocytes (MKs) in the bone marrow and differentiate from pluripotent stem cells in hematopoietic tissues. The primary function of platelets is coagulation and hemostasis; once blood vessel injury occurs, platelets are rapidly activated, adhere to the wound, and aggregate to form a platelet clot. As a result, they are known as the “band-aids” of the bloodstream. Platelets play an executive role in the clinical treatment of blood diseases, such as acute myeloid leukemia, immune thrombocytopenia, and idiopathic thrombocytopenic purpura [1]. Platelets are overlooked immune regulators; they play significant roles in inflammation and infection [2] as they can recognize exterior pathogens and produce many chemoattractants to activate and recruit leukocytes into the site of infection and inflammation, thereby enhancing their lethality to pathogens [3].

The roles of platelets in assisting liver regeneration, bone regeneration, and in the treatment of dermatological conditions, have also increased the demand for platelets in clinical treatment [4–6]. The discovery of platelet-derived serotonin involved in hepatic regeneration and the correlation between impaired platelets and liver cell proliferation suggest that platelets play a significant role in liver regeneration [7, 8]. Platelet transfusion can improve CCl4-induced liver fibrosis in mice with severe combined immune deficiency [9]. The transfer of coding and regulatory RNA between platelets and hepatocytes can promote hepatocyte proliferation and liver regeneration [10–12]. After hepatectomy, platelets coordinate with liver sinusoidal endothelial cells and Kupffer cells via the release of various growth factors, including human growth factor, insulin-like growth factor, and vascular endothelial growth factor (VEGF), or through direct contact with hepatocytes [13–15]. As the therapeutic role of platelets in many diseases is being studied, the application of platelet-rich plasma (PRP) products has gained extensive attention in regenerative medicine. PRP is an autologous biological product derived from centrifuging or apheresis of blood and is a solution with high concentration of platelets [16, 17]. PRP treatment utilizes platelets with abundant biological factors and chemoattractive cytokines associated with tissue regeneration and remodeling.

Moreover, the hydrogel properties of activated PRP make it a suitable medicine delivery vehicle [7, 8, 18]. Platelets dynamically regulate the process of bone remodeling by releasing proinflammatory cytokines to activate the inflammatory phase of early bone healing and then enhance the repair phase of the healing process [19, 20]. PRP treatment has been widely studied in orthopedic and oral/maxillofacial injuries to aid hemostasis and musculoskeletal regeneration [5, 18, 21, 22]. Moreover, in aesthetic dermatology, PRP has been reported to have a therapeutic effect in treating hair loss caused by androgenetic alopecia [23]. Combining platelets with fractional laser or fat grafting can improve scar revision [24, 25] and may provide benefits in skin rejuvenation and dermal augmentation [26, 27]. Thus, platelet therapy is expected to be a new therapeutic avenue for regenerative medicine and tissue engineering.

Previously, donor-derived platelets were the primary platelet source for the treatment of certain clinical diseases such as idiopathic thrombocytopenic purpura (ITP). However, the insufficient supply of donor blood limits its application worldwide. The complexity and doubts surrounding platelet donation have discouraged many donors, and current blood supplies do not meet clinical needs, causing severe shortages [28]. In addition to this problem, there are also several inevitable challenges in platelet transfusion. The first is platelet preservation; platelets can only be stored at room temperature for a short time; otherwise, there is a significant risk of bacterial contamination. Although cold storage can reduce bacterial reproduction and prolong shelf life of the platelets, it also changes platelet structure, molecules, and metabolism [29]. Second, exogenous platelets may cause excessive immune rejection in platelet recipients. Frequent platelet transfusions will cause allogeneic immunity, which results from the generation of multiple antibodies, such as human leukocyte antigen (HLA) antibodies and human platelet antigen antibodies in patients. Residual red blood cells (RBCs) in platelets can also induce RBC antibody production after transfusion [30]. Exploring safe and high-quality alternative sources of platelets for clinical use will markedly benefit the field of regenerative medicine.

Pluripotent stem cells (PSCs), including embryonic stem cells (ESCs) and induced pluripotent stem cells (iPSCs), which have the advantages of unlimited self-renewal and multiple directional differentiation capabilities, have become reliable platelet sources in regenerative medicine. Numerous studies have demonstrated that iPSCs can differentiate into various functional cell types, such as cardiomyocytes, nephron progenitor cells, kidney organoids, oligodendrocyte progenitor cells, and melanocytes [31–34]. Systems for generating induced platelets (iPlatelets) from human PSCs (hPSCs) have also been established using various methods [35–38]. Using gene-editing techniques, such as CRISPR/CAS9, PSCs with great genetic maneuverability can be developed; this makes PSCs more convenient and useful for overcoming some difficulties currently encountered by the use of platelets, such as allogeneic immunity. Therefore, hPSC-derived iPlatelets can overcome the limitations in the current blood donor-dependent system and solve a series of problems in platelet production for clinical application in the near future. However, there are still many challenges to overcome.

This review summarizes current approaches for generating hPSC-derived iPlatelets, presents the current status, compares the advantages and disadvantages, limitations, and defects, and suggests future research direction.

## 2. The Progress and the Current Approaches for iPlatelets

**Figure 1:**
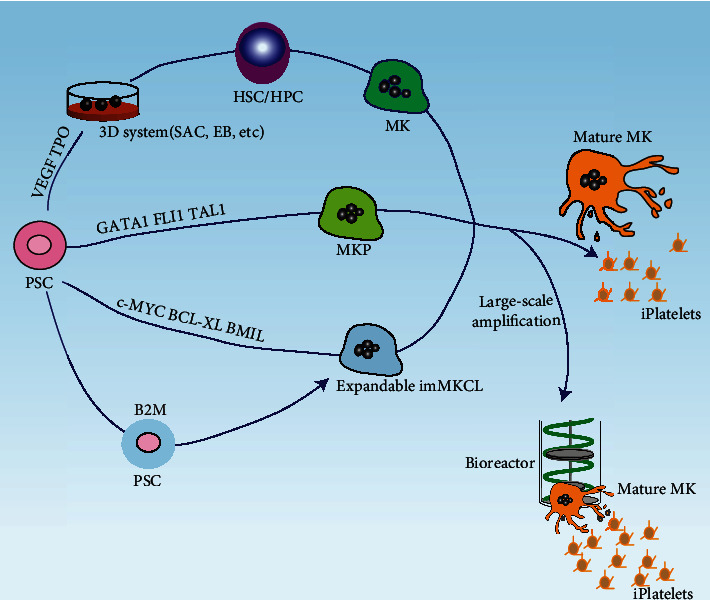
Schema to generate induced platelets from human pluripotent stem cells. HSC/HPC were induced from PSCs via intermediate stage (ESC-sac or EB) under stimulation of multiple cytokines and finally differentiated into mature MK for platelet release. Transgene combination (GATA1/FLI1/TAL1 or C-MYC/BCL-XL/BMI1) provides novel insights for expandable and cryopreserved MK. B2M knockout by CRISPR/Cas9 helped to diminish the allogeneic response caused by HLA mismatch. Furthermore, a 3D bioreactor can be applied for large-scale iPlatelet production. EB: embryoid body; HLA: human leukocyte antigen; HPC: hematopoietic progenitor cell; HSC: hematopoietic stem cell; imMKCL: immortalized megakaryocyte progenitor cell line; MK: megakaryocyte; TPO: thrombopoietin; VEGF: vascular endothelial growth factor.

Many previous studies have reported that MKs are an essential intermediate product during hPSC differentiation into platelets, providing a new perspective for research and blood transfusion medicine. These studies are listed in Table 1; they describe MK differentiation and platelet generation in vitro (Figure 1).

As early as 2006, Gaur and his team established a genetically tractable system to differentiate human ESCs (hESCs) into MKs [39]. A coculture of OP9 stromal cells and hESCs was used to explore MK production in vitro for the first time. It was also called the conventional method or multiround replating method. On days 7 and 11, single cells derived from differentiated hESC colonies were transferred onto fresh OP9 cells and further cultured up to 17 days. Fluorescence analysis showed that approximately 20–60% of floating and loosely adherent cells expressed CD41a/CD42b, characteristic of the megakaryocyte lineage. However, this system yielded few platelets. Furthermore, hematopoietic progenitor cells (HPCs) derived from hPSCs were differentiated into MKs by adding cytokines SCF, Flt3L, and TPO [51, 52]. However, platelet production capacity was still limited. Since then, in attempts to address low yields, different differentiation systems have been established for obtaining hESC-derived platelets. Growth factors such as VEGF and ESC/iPSC-derived sacs (ES/iPS-sacs) differentiated into HPCs, thereby inducing the development of mature MKs and released platelets [35]. In this system, hESCs were cocultured with 10T1/2 or OP9 stromal cells. On day 14 of culture, ES/iPS-sacs were collected to concentrate HPCs, and the latter were then transferred onto fresh 10T1/2 or OP9 stromal cells and cultured up to day 26. With this protocol, a large number of mature platelets were efficiently obtained.

Another successful system was established by Lu et al. who found that hemangioblasts/blast cells (BCs) acted as intermediates in hPSC-derived platelet production and further produced functional MKs on a large scale [40]. In detail, hESCs were cultured in ultra-low attached plates with the addition of multiple cytokine combinations, including BMP4, VEGF, SCF, TPO, and FLT3L, in a serum-free medium for four days. This process involved the embryoid body (EB) formation, which showed excellent potential for industrial iPlatelet production and improvement in differentiation efficiency [40, 53]. The single cells generated from the EB stage were cultured in serum-free medium with multiple cytokines added for blast colony formation on day 8 and further differentiated into MKs in the presence of SCF, TPO, and IL-11 up to days 10–14. For platelet generation, OP9 stromal cells and cytokines, including SCF, TPO, and heparin, were used up to day 22 [40]. The number of platelets derived from these methods is considerable. It has been demonstrated that platelets generated from hESC-derived MKs displayed comparable ultrastructure and morphology with natural platelets and had similar characteristic properties to those of functional platelets [54, 55].

Since the feeder cells and serum addition used in the above culture systems have potential risk for introducing foreign pathogens and induce an immunogenic response in patients, substituting feeder cells and serum was researched. Salvagiotto et al. used collagen IV and a serum-free medium to induce feeder-free iPSCs into early hematopoietic progenitors, displaying the MK lineage characteristics [36]. Further technological innovation, a “spin embryoid body” method, was established for hESC differentiation towards the MK lineage in feeder-free and serum-free differentiation medium [41]. In this protocol, hESCs were cultured in a serum-free medium supplemented with BMP4, VEGF, SCF, and FGF2 to form EBs and commit cells to hematopoiesis. The cells were cultured for an additional 3–10 days in a serum-free medium with TPO, SCF, and IL-3 to stimulate megakaryopoiesis [41]. This serum- and feeder-free culture system enabled the formation of MK progenitors generated from hPSCs and then that of induced platelet-like particles; however, the platelet yield was not provided in this method.

As mentioned earlier, iPSCs provide a promising opportunity to study the ontogeny of hematopoiesis; however, xeno components, such as feeder cells or serum from foreign species, limit the clinical application of iPlatelets. Therefore, Lanza and his team established a three-step protocol that may provide scalable and fully functional platelets for clinical use [38]. This protocol was performed under feeder-free and xeno-free culture conditions, significantly reducing the contamination and immunogenicity caused by foreign serum. Human iPSCs (hiPSCs) were suspended and seeded on human collagen IV-coated plates and incubated for 24 h at 37°C under normoxic conditions in a specific medium. The next day, the cells were transferred to hypoxic conditions and cultured for 4 days, followed by normoxia. The medium contained BMP4, VEGF, and FGF. The cells were cultured in a medium containing TPO, SCF, FLT3L, IL-3, IL-6, and heparin for up to 7 days to generate MK progenitors (MKPs). Finally, MKPs were collected and cultured in an MK maturation medium containing TPO, SCF, IL-6, IL-9, and heparin in ultra-low attachment plates at 39°C for MK maturation and platelet formation. This feeder-free system produced approximately 30 platelets per iPSC-derived MK [38]. In this culture method, proportion of CD41a^+^CD42b^+^ double-positive mature MKs was as high as 80%. The subsequently produced platelets were of high purity, with a further advantage that MKP cells can be cryopreserved [38].

With the rapid breakthrough in technological manipulation, the safety and purity of platelets have improved. At the same time, a method for stable output still needs to be explored for clinical or commercial use. These developed protocols suggest that feeder cells are dispensable for platelet generation in vitro and highlight the importance of media supplements and matrix optimization for future investigation.

### 2.1. Key Genetic Factors during iPlatelet Generation

In addition to improving the differentiation methods mentioned above, specific genetic modifications directly affect platelet production. Previous studies have demonstrated that various tissue lineages can be differentiated from hPSCs by regulating the expression of master transcription factors [56]. Therefore, by manipulating the gene expression of hiPSCs, Takayama et al. found that the expression pattern of *c-Myc* was a crucial factor in determining platelet production during hiPSC differentiation into MKs in vitro [57]. Transient activation of *c-Myc* followed by *c-Myc* expression reduction was critical for MK maturation and functional platelet production. Without this intervention, the constant expression of *c-Myc* decreased the expression of *GATA1* and nuclear factor erythroid-derived 2 p45 unit (p45NF-E2), leading to impaired production of functional platelets [57, 58]. In 2014, Igor Slukvin and his team found that transcription factors *GATA2* and *TAL1* induced hESC differentiation into designated erythrocyte MKPs through hemogenic endothelium intermediates [43]. Two years later, Moreau and his colleagues found that the overexpression of *GATA1*, *FLI1*, and *TAL1* promoted the proliferation and differentiation of MKs. Very high cell yields and purity of MKs could be achieved using entirely chemically defined nonheterogeneous culture conditions [45].

Similarly, immortalized megakaryocyte progenitor cell lines (imMKCLs) were produced after the overexpression of exogenous genes *BMI1*, *BCL-XL*, and *c-MYC* under the control of the Tet-on system in PSCs, and these imMKCLs could expand for a long time and be frozen and thawed [44]. Although these methods involving viral transduction may have specific safety concerns with regards to clinical-grade manufacturing, genetic manipulations offer novel insights into the mechanisms behind megakaryopoiesis; to help develop clinically safe protocols, nonviral or nonintegrated methods or alternative small-molecule compounds should be used in the future.

CD42b (GPIba) is a key marker of functional platelets that can bind to vWF and then initially mediate the adhesion of circulating platelets to an injured site [59, 60]. A study at the Children's Hospital of Philadelphia showed that the expression of CD42b was associated with MK maturation [48]. MKs can be classified into three subsets: low granular MKs (LGMKs), high granular MKs (HGMKs) with CD42b expression, and HGMKs without CD42b expression. A gradual decrease in the percentage of LGMKs and HG/CD42b^+^MKs and accumulation of apoptotic HG/CD42b^−^MKs indicates a reduction in platelet yield, and apoptosis inhibition plays a protective effect on MK apoptosis and CD42b exfoliation. The same study also found that HG/CD42b^+^MKs were more likely to induce a response in platelet activators. These MKs may be close to the peak of maturation and have the ability to endocytose coagulation factor FV into alpha-granules. Interestingly, CD42b^high/^FV^+^ MKs represent a subpopulation of HG/CD42b^+^ MKs with larger size and granularity; this indicates that FV uptake may be one of the final markers of the full MK maturity. FV-labeled MKs can release functional platelets after infusion in immunodeficient NOD/Shi-scid/IL-2R*γ*^null^ (NSG) mice, and the same phenomenon can be found in iPSC-derived MKs [45]. Besides, the uptaken FV platelets have increased clot formation and aggregation ability compared to platelets without FV. These results provide new insight into the specific stage of MK maturation and show an experimental basis for PSC differentiation into platelets. FV labeling could be a useful tool to screen mature HG/CD42b^+^MKs during iPlatelet generation and promote high platelet yield if further combined with bioreactors [42, 61].

Since mismatched HLA antigens are one reason for platelet transfusion failure, a new system was constructed to solve alloimmunity. Using the CRISPR/Cas9 technology to knock out the expression of the *β*2-microglobulin gene (*B2M*), an essential component of HLA class I molecules, the HLA I expression on the cell surface was successfully eliminated [38, 50]. As a result, the functional platelets produced could escape antibody-mediated cytotoxicity both in vitro and in vivo.

### 2.2. Scale-Up System for iPlatelet Production

The efficiency of platelet production from iPSCs in vitro under static culture conditions is lower than that observed in vivo. Many bioreactors or novel culture systems have been developed and refined to scale up iPSC-derived platelet production. A two-dimensional flow culture system was proposed by Nakagawa et al. [42], and it was a biomimetic artificial vascular system consisting of a biodegradable scaffold with an ordered array of holes that were arranged to mimic bone marrow in vivo through salt leaching. In the method, two different flows (the angle between them was 60° instead of 90°) helped to apply appropriate pressure and shear stress to the MKs, thereby promoting platelet production. Platelets derived from hESCs or hiPSCs at a 60° angle through this bioreactor showed complete integrin *α*IIb*β*3 activation after agonist stimulation.

A rotary cell culture system was developed and applied to potentiate megakaryopoiesis, which significantly enhanced platelet generation [62]. This 3D dynamic culture system has advantages in supplying shear force, simulated microgravity, and better diffusion of nutrients and oxygen. By screening chemical compounds, growth factors, and the rotary suspension culture system, the platelet yield was ∼3.7-fold higher than that under static conditions.

Interestingly, turbulence energy, which acts as a physical regulator in thrombopoiesis in vivo, can be applied in platelet production ex vivo. Ito et al. [49] successfully generated highly efficient iPSC-derived platelets using another newly developed vertical reciprocal motion liquid culture bioreactor, VerMES. An optimal level of turbulent energy and shear stress was included in this system, which improved the platelet yield for the generation of 100 billion functional platelets from hiPSC-MKs in an 8 L VerMES independent of the cultivation scale size. The morphology and function of iPSC-derived platelets were comparable to those of donor-derived platelets. The possible explanation was that turbulent energy might promote proplatelet shedding via a cell-autonomous mechanism by soluble factors IGFBP2, MIF, and NRDC [49].

The platelet tracer technology has been further refined. It was found that the level of microtubule component molecule *β*1-tubulin (TUBB1), which is the main component of the cytoplasm of MKs, gradually increases with the maturation of MKs. Therefore, using CRISPR/Cas9 in *β*1-tubulin tagging can help monitor MK development and the generation of platelet-like particles. CRISPR/Cas9 can be applied to the high-throughput identification and validation of novel inducers of large-scale ex vivo platelet production [63]. These highly efficient and controllable methodologies represent a considerable leap in large-scale platelet production for future biomedical and clinical applications.

### 2.3. Functionality of iPlatelets

Many studies have been designed to evaluate whether hPSC-derived MKs and platelets have good purity and appropriate quality as normal human platelets and identify their essential functions both in vitro and in vivo. The assessment criteria for in vitro functionality and safety validation of platelets include, but are not limited to, morphology, ultrastructure, cytoskeletal organization, granule content, ploidy, gene expression, and biomarker expression [64]. Ultrastructural/morphological analyses showed that imMKCLs or other iPSC-derived MKs had similar polyploid states as primary human MKs [44]. iPlatelets have similar surface markers as blood-derived platelets; however, they have fewer platelet granules and larger cell sizes [38, 44]. iPSC-derived MKs expressed many functional specific markers such as CD41a, CD42b, and CD61, which can be used for platelet identification [35, 41]. Further, the relative expression of megakaryocytic marker gene mRNAs, such as *c-Myc*, *GATA1*, *TAL1*, and *RUNX1*, was higher in hPSC-derived MKs [41, 43, 44]. Typically, platelets show aggregated responsiveness after activation by the agonist adenosine diphosphate (ADP) because there are sites on platelets that can bind to ADP and cause a conformational change in integrin *α*IIb*β*3 [35]. Flow cytometry analysis showed the high binding ability of iPlatelets to PAC-1 in the absence of ADP. Upon activation, iPlatelets on immobilized fibrinogen exhibited aggregates, lamellipodia, filopodia, and actin stress fibers, which induced cytoskeletal reorganization [44, 57].

Undoubtedly, the strongest evidence comes from in vivo experiments. Human normal platelets and iPSC-platelets were intravenously infused into macrophage-depleted mice to analyze the iPlatelet function in live animals. It was shown that iPSC-platelets were incorporated into developing mouse thrombus, similar to blood-derived platelets [38]. There is also evidence that iPSC-platelet kinetics is the same as that of fresh normal platelets. With high spatiotemporal resolution confocal laser microscopy, the behavior of iPlatelets upon initiation of adhesion to the injured vessel wall was dynamically observed in immunodeficient NSG mice irradiated with 2.0 Gy to induce thrombocytopenia [57, 65]. Several studies have evaluated the in vivo functions of iPlatelets, and it has been shown that iPlatelets adhered to the injured site initially, leading to thrombus formation and clot retraction [38, 40, 45]. However, some of these studies showed a limited iPlatelet thrombosis capacity compared to normal platelets [44, 45, 57].

### 2.4. Advantages, Limitations, and Potential Applications

Due to the unlimited self-renewal capacity of hPSCs, iPlatelets can be an ideal substitution for current donor-dependent systems. Besides, combination with CRISPR/Cas9 or other gene-editing technologies can help reduce alloimmune rejection [38, 45]. In terms of safety, iPSC-derived platelets can be irradiated to eliminate pathogens and other cell contaminations. As an intermediate, imMKCLs can expand for a long time and can also be frozen and thawed for utilization in emergencies [44]. However, there are still limitations to iPlatelet production, such as potential tumorigenicity, low yield, low functionality, and high cost [37, 38, 42, 44, 45]. The advantages and limitations of iPlatelets have been summarized and listed in Table 2.

Lack of proper models largely restricts the exploration of pathogenesis of many diseases. Thus, patient-derived iPSCs can provide great research models that may address the above issue. For example, the induction of PSCs from patients with familial thrombocytopenia/acute myeloid leukemia showed that their ability to differentiate into HPCs and MKs was affected, which is associated with *RUNX1* germline mutations in these diseases [68]. Further, Paris-Trousseau syndrome (PTSx) is caused by the lack of FLI transcription factor, an essential factor in the process of MK differentiation. To better understand the role of *FLI* in this disease, the platelet production ability of iPSCs derived from PTSx patients was compared with that of wild-type iPSCs, and it was suggested that FLI might influence MK clonogenic potential and the production of platelets [69].

Similarly, because MKs are rare in the bone marrow, it is difficult to explore the differences between healthy and pathological states and their effect on platelet production, especially in patients with severe megakaryocytopenia, including congenital amegakaryocytic thrombocytopenia (CAMT). In this case, iPSCs provide a useful model that mimics thrombocytopenia for patients with CAMT. Although these patient-derived iPSCs cannot produce MKs and platelets, the overexpression of MPL can restore their hematopoietic function [70].

## 3. Conclusion and Future Perspectives

Stable and reliable stem cells are ideal sources for advancing fundamental scientific discoveries and cell therapy in the context of megakaryopoiesis and platelet production. Many studies have demonstrated that platelets derived from hPSCs have certain features and functions analogous to normal platelets, such as the *PAC-1* binding activity, the surface marker expression, and adhesion ability.

This review provides an overview of the possibilities and challenges regarding the production and use of hPSC-derived platelets. Firstly, autologous iPlatelet transfer becomes an effective avenue for averting allogeneic immune rejection. Moreover, the latest knockout technology, CRISPR/Cas9, can also effectively diminish alloimmune rejection due to the mismatched HLA. Then, platelet bioreactors are designed to mimic platelet production in vivo, exposing platelet progenitors to the architecture and intravascular shear stresses characteristic of their native microenvironment [61]. The combination of iPSCs and 3D bioreactors is a useful tool for improving platelet yields [49, 66]. Industrialized production of iPSC-derived platelets is an irresistible general trend for clinical application. With continuous efforts, platelets derived from PSCs have remarkable improvement both in quality and quantity; safety assessment and full functionality evaluation of iPlatelet transfusion are still essential, and the cost needs to be controlled. There is still a long way to go for the large-scale production and clinical application of iPlatelets. iPSC-derived platelets need further technological innovation to realize optimization in terms of scalable production and clinical feasibility.

## Figures and Tables

**Table 1:** Summary of current approaches from human PSCs to iPlatelets.

Cell source	Feeder cells	Multiple stages	Intervention factors	Specific markers	Production	Year
hESCs	OP9	MK	TPO	Not reported	Hardly	2006 [39]
hESCs	C3H10T1/2, OP9	HPC, MK	VEGF, TPO, SCF, heparin	CD41a^+^CD42b^+^	48 ± 0.2 platelets/hESC	2008 [35]
hESCs	According to stage	Hemangioblasts/blast, MK	BMP4, VEGF, SCF, TPO	CD41a^+^CD42b^+^	6.7 ± 0.4 platelets/MK	2011 [40]
hESCs	/	HPC, MK	BMP4, SCF, VEGF, FGF2	CD41a^+^CD42b^+^	Not provided	2013 [41]
hPSCs	C3H10T1/2	HPC, MK	HUVECs (2D bioreactor)	CD41a^+^ or CD42b^+^	Higher than static condition	2013 [42]
hiPSCs	/	HPC, HEC, MKP, MK	Multiple cytokines	CD41a^+^CD42b^+^	About 30 platelets/MK	2014 [38]
hPSCs	C3H10T1/2, OP9	HPC	*TAL1*, *GATA2*	Not reported	Not provided	2014 [43]
hiPSCs	C3H10T1/2	imMKCLs	*BMI1*, *BCL-XL*, *c-MYC*	CD41a^+^CD42b^+^	250 MKs/imMKCL	2014 [44]
hPSCs	/	HPC, MK	*GATA1*, *FLI1*, *TAL1*	CD41a^+^/CD42b^+^	About 7 platelets/MK	2016 [45]
hPSCs	/	MK	Shear stress (3D bioreactor)	*β*1-Tubulin1^+^Hoechst^−^CD41^+^CD42b^+^	~42 platelets/MK, ~350 platelets/h	2014 [46] 2016 [47]
hiPSCs	/	HG/CD42b^+^MK	SCF, TPO, IL-9, IL-6	FV^+^CD42b^+^	Not provided	2017 [48]
hiPSCs	C3H10T1/2	imMKCLs	Turbulent flow, shear stress	CD41^+^CD42b^+^	~70–80 platelets/MK	2018 [49]
hiPSCs	C3H10T1/2, OP9	HSC, HPC, MK	*B2M* KO	CD41^+^CD42b^+^	Not provided	2020 [50]

hESCs: human embryonic stem cells; hPSCs: human pluripotent stem cells; hiPSCs: human induced pluripotent stem cells; MK: megakaryocyte; HPC: hematopoietic progenitor cell; HEC: hematopoietic endothelial cell; MKP: megakaryocyte progenitor; HSC: hematopoietic stem cell; imMKCL: immortalized megakaryocyte progenitor cell line; TPO: thrombopoietin; VEGF: vascular endothelial growth factor; BMP4: bone morphogenetic protein 4.

**Table 2:** Advantages and limitations of current approaches for iPlatelet production.

Methods	Advantages	Limitations
OP9/C3H10T1/2 feeder system [39]	The cornerstone of hPSC-MK generation	Low platelet production Long induction period Potential xenogenous contamination
ES-sac system [35]	Identifies most effective cytokines during hPSC-MK generation The basis for efficient production of platelets	Long induction period Potential xenogenous contamination Low platelet production
EB formation system [40, 66]	Improves the efficiency of MK generationbased on the ES-sac system Combined with defined serum- and animal feeder-free conditions Provides evidence for the functionality of iPlatelets in vivo	Limited efficiency in platelet production
Feeder- or serum-free system [36]	Without pathogen contamination	Limited efficiency in platelet production
HLA-universal iPlatelets [38, 50, 67]	Shortens platelet production time Increases MKP yield Reduces the immunoreactivity of iPlatelets	Inevitable off-target effects or genome toxicity effects Limited efficiency in platelet production
imMKCLs [44, 49]	High stability and cryopreserved storage Widely used in future clinical applications Combined with a bioreactor system	High cost The potential risk of exogene integration
Other genetic manipulation [43]	Feasibility in genetic manipulation Discovers new critical factors that determine the fate of iPlatelets	Inevitable off-target effects Exogene integration may have some specific safety concerns in clinical treatment Limited efficiency in platelet production
